# Influence of Enzymatic Hydrolysis and Molecular Weight Fractionation on the Antioxidant and Lipase / α-Amylase Inhibitory Activities In Vitro of Watermelon Seed Protein Hydrolysates

**DOI:** 10.3390/molecules27227897

**Published:** 2022-11-15

**Authors:** Armin Mirzapour-Kouhdasht, Marco Garcia-Vaquero, Jong-Bang Eun, Jesus Simal-Gandara

**Affiliations:** 1 School of Agriculture and Food Science, University College Dublin, Belfield, Dublin 4, D04 V1W8 Dublin, Ireland; 2 Department of Integrative Food, Bioscience and Biotechnology, Chonnam National University, Gwangju 61186, Republic of Korea; 3 Nutrition and Bromatology Group, Department of Analytical Chemistry and Food Science, Faculty of Science, Universidade de Vigo, E32004 Ourense, Spain

**Keywords:** antioxidant, anti-obesity, protein hydrolysates, bioactive peptides, amino acid composition

## Abstract

This study aims to evaluate the potential in vitro antioxidant and anti-obesity activities of watermelon seed protein hydrolysates (WSPH) obtained using different combinations of enzymes alcalase–proteinase K (ALC-PK) and alcalase–actinidin (ALC-ACT). There was a direct relationship between the degree of hydrolysis (DH) and the biological activities of the WSPH, with the highest DPPH (approximately 85%) and lipase inhibitory activities (≈59%) appreciated at DH of 36–37% and 33–35% when using ALC-PK and ALC-ACT, respectively. Following molecular weight fractionation, the ALC-PK WSPH < 3 kDa (F1) assayed at 1 mg.mL^−1^ had the highest DPPH-radical scavenging (89.22%), ferrous chelating (FC) (79.83%), reducing power (RP) (A 0.51), lipase inhibitory (71.36%), and α-amylase inhibitory (62.08%) activities. The amino acid analysis of ALC-PK WSPH and its fractions revealed a relationship between the biological activity of the extracts and their composition. High contents of hydrophobic amino acids, arginine, and aromatic amino acids were related to high antioxidant, lipase inhibitory, and α-amylase inhibitory activities in the extracts, respectively. Overall, this study revealed that underutilized protein sources such as WSPH, using the appropriate combination of enzymes, could result in the generation of new ingredients and compounds with powerful antioxidant and anti-obesity activities with promising applications as nutraceuticals or functional foods.

## 1. Introduction

The interest in food-derived protein hydrolysates has recently increased in the scientific literature due to the potential health benefits and, thus, potential applications of these hydrolysates for their use as functional foods or nutraceuticals helping in the prevention of several disorders [1]. Amongst their multiple potential health benefits and applications, protein hydrolysates have gained relevance as antioxidant [2,3,4], antidiabetic [5,6], antihypertensive [7,8,9], anti-obesity [10,11,12], and antimicrobial compounds [13,14,15,16]. Moreover, several of these peptides may exert multiple health benefits, and thus, the relevance of protein hydrolysates with multifunctional properties can be of particular importance when fighting multiple health disorders.

Moreover, synthetic drugs used to treat or fight several diseases can be linked to undesirable side effects. For example, the inhibition of α-amylase and lipase by synthetic drugs, such as acarbose and sibutramine, is currently considered a prominent strategy to fight against obesity. However, the use of these drugs is currently linked to some undesirable and inevitable side effects, including flatulence, diarrhea, and abdominal pain [17]. The discovery and use of novel natural compounds as alternatives to synthetic drugs and their side effects have promising potential [18,19].

*Citrullus lanatus* L. (watermelon) is a member of the Cucurbitaceae family [20]. The protein content of seeds in this species has been reported to be around 30 to 43% [21], constituting an underutilized source of protein, opening huge commercial and potential applications of these proteins for food and health-related industries. Moreover, the hydrolysis of protein substrates by consecutive action of different proteases is considered one of the most effective procedures to obtain bioactive peptides with specific biological activities [22,23]. The hydrophobic amino acid residues in the watermelon seed protein are potential cleavage sites for alcalase and proteinase K hydrolysis (ALC-PK), and previous results have shown the potential of the hydrolysis of proteins with alcalase and actinidin (ALC-ACT) for generating bioactive peptides [9].

Since all countries in the world pose environmental concerns, managing food waste is a major issue. A way to reduce food waste and its negative effects on the environment is to employ these byproducts in the manufacturing of products in a number of sectors, including food, pharmaceuticals, and cosmetics. Additionally, this article highlighted more than one in vitro mechanism of anti-obesity as well as antioxidant activities of the watermelon seed protein hydrolysates (WSPH) to show the right path for further expansion to in silico, ex vivo, and in vivo experiments. On the other hand, using ultrafiltration with different MW cut-offs helps to focus on a narrow range of hydrolysates to save time and consumption of laboratory materials and find the best fraction with the highest efficiency against chronic diseases in question.

This study explored the use of both ALC-PK and ALC-ACT hydrolysis procedures as a promising strategy to produce WSPH containing multifunctional peptides. The potential antioxidant (measured as DPPH, ferrous chelating (FC), reducing power (RP) activities), and anti-obesity activities (lipase and α-amylase inhibitory activities) of the full hydrolysate and different molecular weight fractions was also investigated together with the amino acid composition of the most active hydrolysates generated from WSPH.

## 2. Results and Discussion

### 2.1. Degree of Hydrolysis

**Figure 1:**
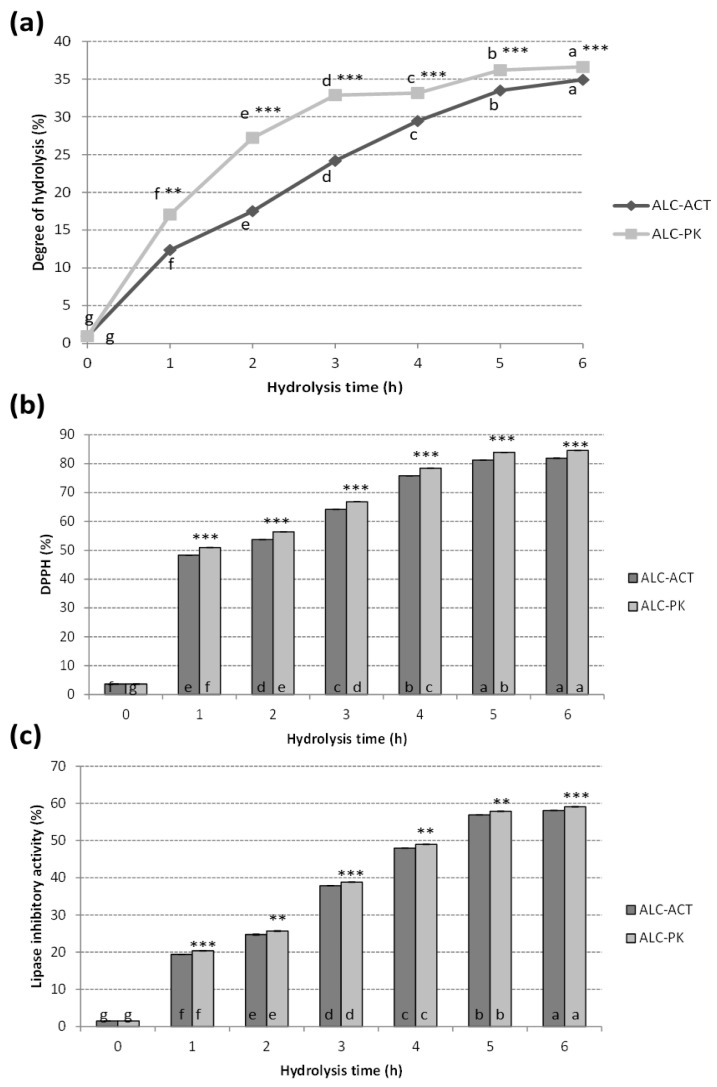
Representation of (**a**) the degree of hydrolysis (DH) of watermelon seed proteins following combination of enzymes alcalase–proteinase K (ALC-PK) and alcalase–actinidin (ALC-ACT) monitored at hourly (0–6 h). Data are presented as the average of three replicates. The biological activities of these hydrolysates at different times of hydrolysis for both ALC-PK and ALC-ACT processes are also summarized in (**b**) DPPH and (**c**) lipase inhibitory activity. Data are presented as the average ± standard deviation of the mean of three replicates. Different letters represent statistical differences (*p* < 0.05) between different times of hydrolysis within the same enzymatic treatment (lowercase letters). Statistical differences between enzymatic treatments (ALC-PK and ALC-ACT) at the same hydrolysis time are represented as ** *p* < 0.01, *** *p* < 0.001.

The DH of proteins is an important parameter as it may influence the biological properties of the resultant hydrolysates. The molecular sizes and amino acid contents of the peptides can be affected by DH, which has a significant impact on the biological activities of the peptides [24]. The results of DH for both extraction procedures using the enzyme combinations ALC-ACT and ALC-PK are presented in Figure 1a. From the initiation of the enzymatic reaction, the ALC-PK hydrolysates showed a statistically (*p* < 0.01 for 1 h and *p* < 0.001 for further hydrolysis times) higher DH in comparison with ALC-ACT hydrolysates. For both hydrolysates, the DH values increased with a direct relationship to the hydrolysis time. The DH at 5 and 6 h for ALC-PK hydrolysates were 36.18 and 36.60%, respectively, while the DH of the ALC-ACT hydrolysates was 33.48% (5 h) and 34.90% (6 h). The direct and non-linear relationship between DH and hydrolysis time shows that ALC-ACT and ALC-PK hydrolyzed over 84% and 90% of the protein during the first 4 h of the reaction, respectively. Compared to the first 4 h of reaction, the DH experiences continued to increase at a slow rate. These results are in agreement with previous research analyzing the hydrolysis process of skate skin gelatin [25]. The authors reported that a combination of subtilisin and actinidin hydrolyzed 80% of the gelatin during the first 4 h of the reaction. Selected antioxidant and anti-obesity activities in vitro (DPPH and lipase inhibitory activities) were also monitored at various hydrolysis times to gain an insight into the effect of the hydrolysis process on the main activities analyzed while minimizing the uptake of sample from the reaction vessels that could negatively affect the hydrolysis process (Figure 1b,c). The DPPH and lipase inhibitory activities of ALC-PK for 5 and 6 h of reaction were as follows: 83.91 and 84.57%, 57.88 and 59.09%, respectively. The DPPH and lipase inhibitory activities achieved by the hydrolysates using ALC-ACT for 5 and 6 h of reaction were as follows: 81.24 and 81.90%, 56.90 and 58.11%, respectively. Typically, a single enzyme cannot perform considerable hydrolysis as efficiently as an enzyme combination. In addition, consecutive enzyme reactions can result in a considerable increase in the biological activities of the resulting hydrolysates. Moreover, the use of different enzymes and combinations of them during these hydrolytic processes can result in remarkable differences in the biological activities of the final products [26]. However, there are also conflicting results in the literature, as some studies indicated that the use of combined enzymes had no positive impact on the antioxidant activities of the final products [27]. As the differences in biological activities of the hydrolysates generated at 5 and 6 h were minimum, 5 h hydrolysates were the ones considered for further exploration to reduce the time of the reactions and chemicals used during this procedure, aligning with current sustainability trends in the food industry [28].

Overall, the DH showed a direct relationship with biological activities. These results were in agreement with previous studies on protein hydrolysis using ALC-ACT [9]. Moreover, the optimum hydrolysis time of the proteins in this study was also in agreement with previous reports. Jafar et al. [29], when hydrolyzing camel whey protein with pepsin, trypsin, and chymotrypsin, appreciated that pepsin and chymotrypsin hydrolysates after 6 h of enzymatic reaction were the most active samples considering both DH and lipase inhibitory activity compared to the other procedures analyzed.

### 2.2. Antioxidant Activity

#### 2.2.1. DPPH-Radical Scavenging Activity

**Figure 2:**
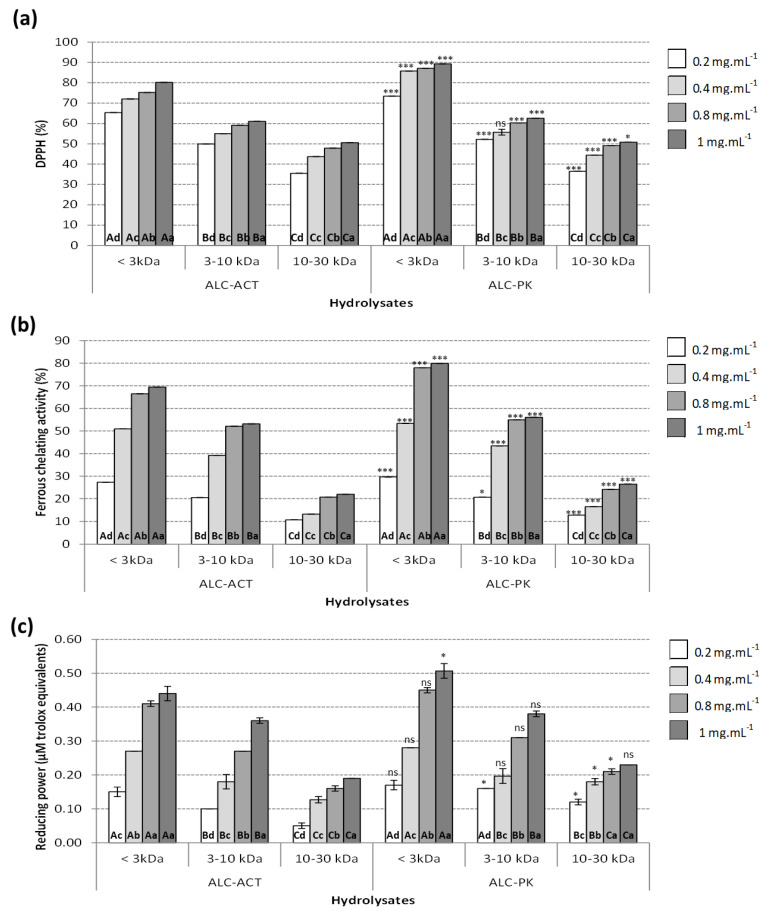
Antioxidant activity measured as (**a**) DPPH (%), (**b**) ferrous chelating activity (%) and (**c**) reducing power (mM trolox equivalents) of alcalase–actinidin (ALC-ACT) and alcalase–proteinase K (ALC-PK) watermelon seed hydrolysates ultrafiltered fractions (<3 kDa, 3–10 kDa, and 10–30 kDa) tested at different concentrations ranging from 0.2 to 1 mg.mL^−1^. Data are presented as average ± standard deviation (*n* = 3). Different letters inside the figure represent statistical differences (*p* < 0.05) in activity. Different lowercase letters inside the figure refer to differences in activity between the different concentrations tested, using the same enzyme and molecular weight fraction. Different uppercase letters inside the figure refer to differences in activity between different molecular weight fractions using the same enzymes and concentrations. Statistical differences between enzymatic treatments (ALC-PK and ALC-ACT) at the same hydrolysis time and concentrations are represented as ns (non-significant), * *p* < 0.05, ** *p* < 0.01, *** *p* < 0.001.

The DPPH radical scavenging activity of ALC-ACT and ALC-PK hydrolysates for different MWCO fractions (<3 kDa, 3–10 kDa, and 10–30 kDa) are represented in Figure 2a. For both ALC-ACT and ALC-PK hydrolysates, the DPPH radical scavenging was reversely related to the MW with the highest DPPH appreciated in hydrolysates of <3 kDa, followed by fractions of 3–10 kDa and lastly those of 10–30 kDa. These results contradict previous reports using ALC-ACT when hydrolyzing gelatin [30,31]. These differences could be attributed to differences in the protein substrates and amino acid composition between studies. Several studies also revealed a reverse relationship between the MW and DPPH radical scavenging activity of protein hydrolysates [32,33,34]. The DPPH radical scavenging activity of the hydrolysates generated using ALC-PK had similar behavior to the ones described for ALC-ACT. The highest DPPH radical scavenging was achieved by the <3 kDa hydrolysates when tested at 1 mg.mL^−1^ (89.22%). Moreover, for all the ALC-ACT and ALC-PK hydrolysate fractions tested, the DPPH radical scavenging activity had a dose-dependent manner when tested at concentrations ranging from 0.2 to 1 mg.mL^−1^, always reaching maximum DPPH activities at concentrations of 1 mg.mL^−1^. Several studies demonstrated that the DPPH radical scavenging activity is indirectly related to the MW of peptides and protein hydrolysates [35,36,37,38]. However, this is not the only factor influencing DPPH radical scavenging activities. The amino acid composition of hydrolysates is one of the most prominent influencing parameters affecting the DPPH radical scavenging activity of the compounds [37]. Due to the significantly higher biological activities of ALC-PK hydrolysates compared to those of ALC-ACT, the amino acid composition of ALC-PK hydrolysates was determined (Table 1) to investigate its relationship with antioxidant and anti-obesity activity. The higher DPPH radical scavenging activity of hydrolysates of low MW (<3 kDa) could be related to its higher contents of hydrophobic amino acids such as alanine, phenylalanine, isoleucine, methionine, and valine as the DPPH radical is a hydrophobic substance that interacts well in systems with hydrophobic components [39]. The same conclusions were reached by Mundi and Aluko [40] while examining the biological effects of kidney bean protein hydrolysates. The authors demonstrated that the high DPPH inhibitory activity of some hydrolysate fractions was related to the high hydrophobic aromatic (phenylalanine and tyrosine) and hydrophobic aliphatic (valine, isoleucine, and leucine) amino acid residue contents of these fractions. Another study by Pownall et al. [41] reported that fractions of pea seed protein hydrolysate that eluted last when separated via reverse-phase high-performance liquid chromatography had a higher DPPH inhibitory activity compared to the fractions eluted first. The authors also associated the high DPPH activity of these fractions with their high contents in leucine, phenylalanine, valine, and tryptophan, which also influenced the elution pattern of these compounds.

#### 2.2.2. Ferrous Chelating (FC) Activity

FC activity is regularly performed to evaluate the antioxidant activity of bioactive compounds derived from protein hydrolysates, and this activity is influenced by the MW of the compounds [42]. The lipid peroxidation process, which forms the hydroxyl radical (OH-) and increases the Fenton reaction, is initiated and propagated by the ferrous ion, which is a transition metal. Consequently, the chelation of ferrous ions serves as both primary (prevents the development of lipid peroxidation) and secondary (prevents chain oxidation) antioxidants. When ferrozine substantially creates a complex with ferrous in which the red color is diminished due to the presence of chelating compounds, the chelation reaction is started [43].

The FC activity of ALC-ACT and ALC-PK hydrolysates and their fractions are represented in Figure 2b. The highest FC of both ALC-ACT and ALC-PK hydrolysates was achieved by the fraction < 3 kDa tested at 1 mg.mL^−1^ with FC levels of 69.44 and 79.83%, respectively. Similar to the case of DPPH, the order of FC activity in UF fractions were as follows: F1 (<3 kDa)> F2 (3–10 kDa) > F3 (10–30 kDa). Farvin et al. [44] also appreciated that protein hydrolysates from *Gadus morhua* with a low MW (<3 kDa) had higher FC compared to higher MW fractions when tested at the same concentrations.

There are studies in which the indirect relationship between FC and MW of bioactive peptides was revealed. As reported by Wu et al. [45], bioactive peptides derived from different protein origins with MW between 0.3 kDa and 1.5 kDa usually have a strong affinity to iron, a fact that could explain the high FC activity of the F1 (<3 kDa) of the hydrolysates generated from WSPH in the current study.

#### 2.2.3. Reducing Power (RP) Activity

The RP of a bioactive compound is directly related to its antioxidant activity [46,47]. The results of the RP activity of ALC-ACT and ALC-PK hydrolysates for the different MWCO fractions are represented in Figure 2c. RP followed the same pattern as that described previously for FC with the highest RP activities for both ALC-ACT and ALC-PK hydrolysates at 1 mg.mL^−1^ in F1 (<3 kDa) of 0.44 and 0.51 mM trolox equivalents, respectively. No significant differences were also appreciated when assaying the ALC-ACT hydrolysates < 3 kDa and the ALC-PK hydrolysates 10–30 kDa at 0.8 or 1 mg.mL^−1^; however, significant differences were appreciated between both concentrations in the remaining hydrolysates tested. Similar to the other activities analyzed, the RP activity ordered from high to low of the UF fractions was as follows: F1 (<3 kDa) > F2 (3–10 kDa) > F3 (10–30 kDa). Previous research identified that the relative abundance or presence of certain amino acids, such as leucine, specifically affected the RP activity of the peptides [48], which may also explain the results of the current study for the high RP activity of the F1 fraction. The significantly higher leucine content in F1 (7.18 ± 0.01 leucine in 100 amino acid residues) confirms the abovementioned state (Table 1). Safari and Yaghoubzadeh [49] demonstrated that the RP of *Holothuria leucospilota* protein hydrolysates increased following the MWCO of the hydrolysates, similar to the results achieved by other studies on the RP activity of rice bran protein hydrolysates [50]. The latter authors demonstrated that the fraction with an MW < 4 kDa had a higher RP in comparison with those fractions with higher MW.

### 2.3. Anti-Obesity Activities

#### 2.3.1. Lipase Inhibitory Activity

Pancreatic lipase is an important enzyme affecting the absorption of neutral lipids (triglycerides) in the small intestine, and thus, the use of peptides that could actively inhibit the activity of this enzyme could be a promising strategy for the prevention of obesity and hyperlipidemia. To date, few studies have explored the lipase inhibitory potential of plant-derived protein hydrolysates [51].

**Figure 3:**
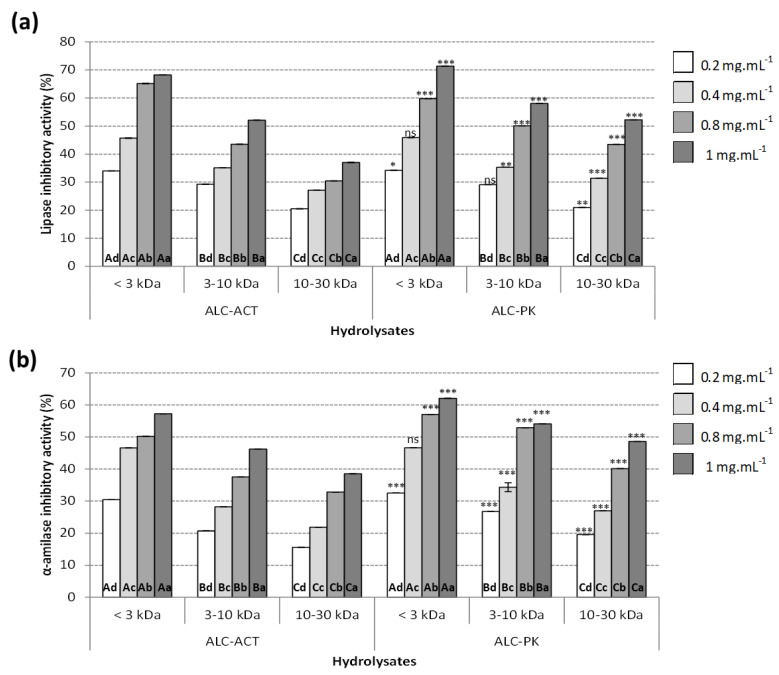
Anti-obesity activity measured as (**a**) lipase inhibitory activity (%) and (**b**) α-amylase inhibitory activity (%) of alcalase–actinidin (ALC-ACT) and alcalase–proteinase K (ALC-PK) watermelon seed hydrolysates ultrafiltered fractions (<3 kDa, 3–10 kDa, and 10–30 kDa) tested at different concentrations ranging from 0.2 to 1 mg.mL^-1^. Data are presented as average ± standard deviation (*n* = 3). Different letters inside the figure represent statistical differences (*p* < 0.05) in activity. Different lowercase letters inside the figure refer to differences in activity between the different concentrations tested, using the same enzyme and molecular weight fraction. Different uppercase letters inside the figure refer to differences in activity between different molecular weight fractions using the same enzymes and concentrations. Statistical differences between enzymatic treatments (ALC-PK and ALC-ACT) at the same hydrolysis time and concentrations are represented as ns (non-significant), * *p* < 0.05, ** *p* < 0.01, *** *p* < 0.001.

In the current study, the lipase inhibitory activity of different fractions was determined based on the measurement of the release of 4-nitrophenol, as represented in Figure 3a. The lipase inhibitory activity of the hydrolysates of the current study was inversely related to the MW of protein hydrolysates. In general, ALC-PK hydrolysates had higher lipase inhibitory compared to the ALC-ACT hydrolysates at most concentrations tested. For both ALC-PK and ALC-ACT hydrolysates, the lipase inhibitory activity was dose-dependent and positively related to the hydrolysate’s concentration. The highest achieved lipase inhibitory values for ALC-PK and ALC-ACT hydrolysates were 71.36 and 62.08% (at 1 mg.mL^−1^), respectively. These results were in agreement with previous research performed by Ketprayoon et al. [52] on de-oiled rice bran protein hydrolysates obtained by Alcalase^®^. The authors fractionated these hydrolysates, and the lipase inhibitory activity of the hydrolysates was also inversely related to the MW of the compounds. However, the underlying mechanisms of action of the lipase inhibitory activity of protein hydrolysates have not been fully elucidated yet. Siow et al. [53,54] reported that cumin seed peptides hindered the catalytic activity of the lipase enzyme through the direct attachment of these peptides to the active enzymatic sites. Similarly, Martinez-Villaluenga et al. [55] revealed that the lipase inhibitory activity of soybean peptides was achieved by interacting with the catalytic site of the lipase enzyme following a similar mechanism of action of well-known synthetic anti-obesity drugs, such as Orlistat.

Moreover, and similarly to the present study, the lipase activity of peptides was also linked to their composition and particularly the presence of arginine, as the presence of this amino acid in bioactive peptides could promisingly increase their lipase inhibitory activity [56]. This may also explain the results of the current study, as the content of arginine in the different fractions of the hydrolysate with the highest lipase inhibitory activity (ALC-PK) (Table 1) followed the same pattern as those of their lipase inhibitory, with the highest Arg and lipase inhibitory activities described in F1 (<3 kDa) followed by F2 (3–10 kDa) and lastly F3 (10–30 kDa).

#### 2.3.2. α-Amylase Inhibitory Activity

The inhibition of α-amylase aims to decrease the blood glucose level [57], and thus, novel compounds with α-amylase inhibitory activity could be an efficient strategy to control obesity and type II diabetes when included in the diet via the development of functional foods.

The results of the α-amylase inhibitory activity of hydrolysates generated in this study are shown in Figure 3b. Similar to the case of lipase inhibitory activity, the α-amylase inhibitory activity of the hydrolysates studied was inversely related to the MW of the hydrolysates. The ALC-PK hydrolysates had statistically (*p* < 0.05) higher α-amylase inhibitory compared to the ALC-ACT hydrolysates at all concentrations. Both ALC-PK and ALC-ACT hydrolysates exhibited dose-dependent α-amylase inhibitory activity with the highest activities of 62.08% inhibition in the case of ALC-PK, and 57.19% inhibition ALC-ACT both assayed at 1 mg.mL^−1^. These results are similar to those described by Ngoh and Gan [58], who associated the highest α-amylase inhibitory with the low MW fractions achieved from protein hydrolysates from *Phaseolus vulgaris* cv, attributing this effect to the amino acid composition of the fractions. Further studies also established that some amino acids are able to establish hydrogen bonds with other amino acid residues in the active site of the α-amylase enzyme. Particularly, the aromatic residues of the active site of α-amylase can interact with bioactive peptides containing aromatic amino acids, such as phenylalanine and tyrosine. Therefore, hydrogen bonds alongside electrostatic and Van der Waals interactions are responsible for peptide–α-amylase aromatic interactions resulting in α-amylase inhibitory [54]. This is in agreement with the amino acid composition of the most active fractions achieved by the hydrolysates generated by ALC-PK. The content of aromatic amino acids was at its highest in F1 (<3 kDa), followed by F2 (3–10 kDa), and the lowest levels in F3 (10–30 kDa), which is also reflected in the α-amylase inhibitory activity of these fractions.

## 3. Materials and Methods

**Figure 4:**
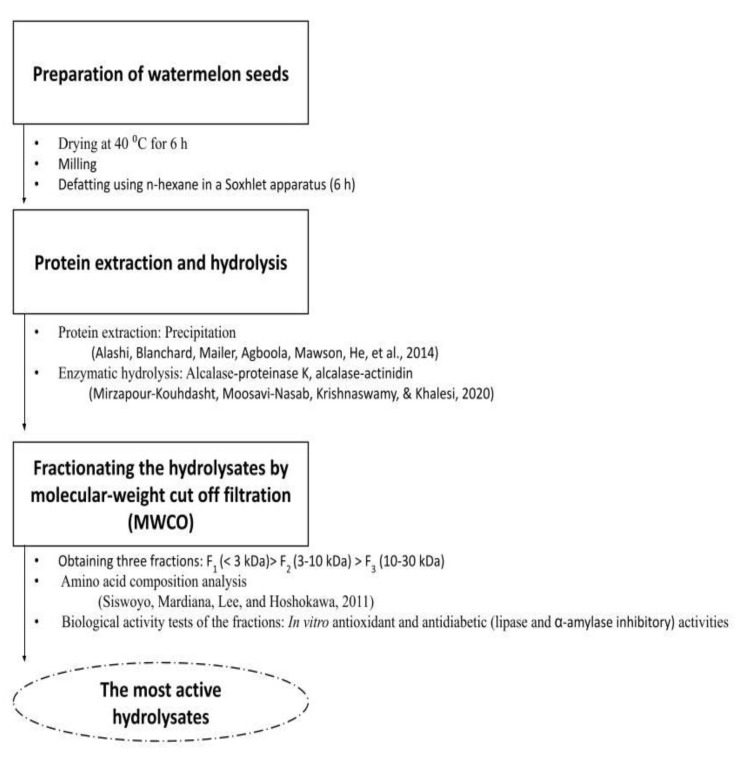
Schematic representation of the procedures used to generate protein hydrolysates from watermelon seed and analyze their biological activities.

The overall process and determinations used in this study are summarized in Figure 4.

### 3.1. Biological Material and Chemical Reagents

Fresh and equal-sized watermelons (*Citrullus lanatus* L.) were purchased from a local farm market in Shiraz, Iran, and immediately transported to the lab in polystyrene storage boxes. Proteinase K from the *Tritirachium album* (≥40 mAnson U.mg^−1^) was provided by Sigma Aldrich, Inc. (St. Louis, MO, USA). Alcalase from *Bacillus licheniformis* (406.80 U.mg^−1^) was provided by the National Institute of Genetic Engineering and Biotechnology (Tehran, Iran). Actinidin (174.26 U.mg^−1^) was prepared from a previous study conducted in the Department of Integrative Food, Bioscience, and Biotechnology, Chonnam National University, Gwangju, South Korea [9]. All the chemicals and reagents were of analytical grade and provided by Sigma Aldrich, Inc. (St. Louis, MO, USA).

### 3.2. Protein Extraction and Hydrolysis

Watermelon seeds were oven dried at 40 °C for 6 h, pulverized, and defatted using n-hexane in a Soxhlet apparatus (6 h), evaporating the solvent in a vacuum rotary dryer (Laborota 4000, Heidolph, Germany). The protein was extracted from the defatted powder following the method described by Alashi et al. [59] with some modifications. Briefly, the defatted powder was suspended in 0.1 M NaOH (pH 12) at a ratio of 1:10 (*w/v*) while stirring for 60 min, followed by centrifugation of the sample at 3000× *g* for 10 min (18 ± 2 °C). These extraction/centrifugation steps were repeated twice, and the resulting supernatants were pooled together. The protein was precipitated by pH shift by lowering the pH of the sample to 4 by adding an acidic solution (1 M HCl). The precipitate was collected by centrifugation following the previously mentioned conditions (3000× *g*, 10 min, 18 ± 2 °C), neutralized, and washed with distilled water before drying by a freeze dryer (IFD-5012, Dena Vacuum, Iran).

The protein extract was hydrolyzed following the procedure described by Mirzapour-Kouhdasht et al. [31] with slight modifications. Briefly, a protein solution (5% *w/v*) was prepared in Tris-Base at optimum pH value for each protocol of hydrolysis (ALC-ACT (8.5–7.5) or ALC-PK (8.5–8.0)). The hydrolysis procedures were performed at an enzyme-to-substrate ratio of 1:100 (*w/w*) for 6 h under continuous stirring (80 rpm), and the pH of the reactions was adjusted during the process with 0.1 N NaOH. The enzymatic reactions were terminated by boiling the solutions for 20 min, centrifuging the solutions (8000× *g*, 15 min, 10 °C), and freeze-drying the supernatants. The protein hydrolysates generated following this procedure were kept at −20 °C in 15 mL centrifugal tubes until further analyses and experimental work.

### 3.3. Degree of Hydrolysis (DH)

The DH of the samples achieved during the 6 h of the hydrolysis reaction was measured at 1 h intervals (0–6 h) as described by Adler-Nissen [60] using the following Equation (1).

DH (%) = h/h_tot_ = (Nb × V × 100)/(α × Mp × h_tot_) (1)

where Nb represents the molarity of the NaOH solution; V is the volume of NaOH used in mL; Mp is the protein mass (g); α is the degree of dissociation of the α-amino groups; and h_tot_ is the total moles of the peptide bond.

### 3.4. Molecular-Weight Cut-Off Filtration (MWCO)

The WSPH was fractionated using 30 kDa, 10 kDa, and 3 kDa MWCO ultrafiltration amicon filter tubes at 4 °C obtaining three fractions, namely F1 (<3 kDa), F2 (3–10 kDa), and F3 (10–30 kDa). All the fractions were freeze-dried and kept at −20 °C until further experiments.

### 3.5. Antioxidant Activity

All the antioxidant activities of the various hydrolysates generated at different hydrolysis times and hydrolysate fractions separated by MWCO were performed in triplicate.

#### 3.5.1. DPPH Radical Scavenging Activity

The DPPH radical scavenging activity of the samples was measured as described by Ambigaipalan and Shahidi [61] with slight modifications. An amount of 200 μL of samples were poured into 800 μL of 0.1 mM DPPH in 95% methanol solution. The mixtures were incubated in dark conditions at 25 °C for 30 min. The absorbance of samples was read at 517 nm in a microplate reader (BioTek Instruments, Winooski, VT, USA). Methanol solution (95%), distilled water, and ascorbic acid were applied as a control sample and negative and positive controls, respectively. The DPPH radical scavenging activity of samples was calculated using the following Equation (2):

DPPH radical scavenging (%) = (Ac − As)/Ac × 100(2)

where Ac and As represent the absorbance of the control samples and tested samples, respectively.

#### 3.5.2. Ferrous Chelating (FC) Activity

The FC activity of the samples was measured following the procedure described by Ambigaipalan et al. [26] with slight modifications. Briefly, 200 µL of tested samples were dissolved in 1.74 mL distilled water. Subsequently, 20 µL of 2 mM FeCl_2_ and 40 µL of 5 mM ferrozine were added, shaking the solutions thoroughly and incubating the reactions at 25 °C for 10 min. For the control, distilled water was applied rather than samples. In order to prepare the blanks, 200 µL of each sample was dissolved in 1.80 mL of distilled water for background subtraction. The absorbance of the solutions was read at 562 nm, and the FC activity was calculated using Equation (3).

FC (%) = [1 − (As/Ac)] × 100(3)

where As and Ac represent the absorbance of the sample and control, respectively.

#### 3.5.3. Reducing Power (RP)

The RP of the samples was measured following the method described by Yen and Chen [62]. Tested samples were added to a 1% potassium ferricyanide solution (1:1 *v/v*) and incubated at 50 °C for 20 min. An amount of 2.5 mL of 10% trichloroacetic acid was later added to the solutions, followed by centrifugation (3000× *g*, 15 min). The supernatant was mixed with distilled water at a ratio of 1:1 (*v/v*), followed by the addition of 0.5 mL of 1% ferric chloride and incubation of the solutions at 25 °C for 10 min. The absorbance of the samples was read at 700 nm. Trolox solutions at concentrations ranging from 0 to 1 mM (0, 0.2, 0.4, 0.6, 0.8, and 1 mM) were used as standard.

### 3.6. Anti-Obesity Activities

The anti-obesity activities of the various hydrolysates generated at different hydrolysis times and hydrolysate fractions separated by MWCO were performed in triplicate.

#### 3.6.1. Lipase Inhibitory Activity

Lipase inhibitory activity of the samples was determined as described by Ambigaipalan et al. [63] with the modifications mentioned by Fathi, Moosavi-Nasab, Mirzapour-Kouhdasht, and Khalesi [19]. Briefly, lipase enzyme (5 mg) was dissolved in 1 M Tris-HCl (pH 8.5). An amount of 100 µL of each tested sample (concentrations ranging from 0.2 to 1 mg.mL^−1^) was added to an equal volume of lipase solution and 4 mL of Tris-HCl buffer (1 M, pH 8.5). The mixtures were incubated at 37 °C for 25 min, and the enzymatic reactions were started by adding 100 µL of the substrate (5 mM palmitate in dimethyl sulfoxide (DMSO): ethanol (at 1:1 *w/v*) to the reaction mixtures following by a second incubation period (37 °C, 25 min). The absorbance of the samples was measured in a microplate reader (BioTek, Winooski, Vermont, US) at 412 nm. The lipase inhibitory activity was determined using the following equation:

Lipase inhibitory (%) = (As − Asb)/(Ac − Acb) × 100(4)

where As, Asb, Ac, and Acb represent the absorbance of the sample, sample blank, control, and control blank, respectively. The blank and control samples were generated by following the aforementioned experimental steps without adding the enzyme or without adding the inhibitor and enzyme, respectively.

#### 3.6.2. α-Amylase Inhibitory Activity

α-amylase inhibitory activity was determined according to the protocol described by Oboh et al. [64]. An amount of 125 µL of sodium phosphate buffer (20 mM, pH 6.9) containing 6 mM NaCl and α-amylase at a final concentration adjusted to 0.5 mg.mL^–1^ was mixed with an equal volume of tested samples at concentrations ranging from 0.2 to 1 mg.mL^−1^. After the pre-incubation of these mixtures (25 °C, 10 min), 125 µL of 1% starch in sodium phosphate solution (20 mM, pH 6.9) containing 6 mM NaCl were added, and the reactions were further incubated at 25 °C for 10 min. The reactions were stopped by adding 250 µL of dinitrosalicylic acid (DNS) and boiling the solutions for 5 min. The mixtures were cooled down and diluted with 2.5 mL distilled water before recording the absorbance at 540 nm. The α-amylase inhibitory activity was calculated using the following equation:

α-amylase inhibitory (%) = (Ac − As)/(Ac) × 100(5)

where Ac and As represent the absorbance of the control and control, respectively. The control was measured by using distilled water instead of tested samples.

### 3.7. Amino Acid Analysis

The amino acid composition of selected hydrolysates based on their biological activities was determined according to the method described by Siswoyo et al. [65], with the modifications previously proposed by Mirzapour-Kouhdasht, Moosavi-Nasab, Krishnaswamy, and Khalesi [31]. Briefly, an aliquot of samples (50 mL) was chemically hydrolyzed using 6 M HCl containing 0.1% phenol at 110 °C for 24 h. Subsequently, the amino acid analysis was conducted using an amino acid analyzer apparatus (HITACHI 8900 Amino Acid Analyzer, Japan). The specifications of this amino acid analyzer unit, as indicated by the manufacturer include a column (4.6 mm i.d. × 60 mm, 3-µm Hitachi Customer Ion Exchange Resin) and column oven (20–85 °C in increments of 1 °C).

### 3.8. Data Analysis

All the biological activities were performed in triplicate. All statistical analyses were performed using IBM SPSS Statistics 23.0 software. The degree of hydrolysis and biological activities over the proposed hydrolysis time were analyzed by repeated measures. The antioxidant and anti-obesity activities of different concentrations of hydrolysates and the differences in the amino acid composition of ALC-PK between full hydrolysate and MWCO fractions were analyzed by a multivariate general linear model. All these differences were further analyzed by either Tukey’s HSD post hoc tests or Student’s *t*-tests. In all cases, the criterion for statistical significance was *p* < 0.05.

## 4. Conclusions

The results of the present study revealed the antioxidant and anti-obesity activities of watermelon protein hydrolysates. Overall, there was a direct relationship between these biological activities investigated and the degree of hydrolysis of the proteins. From the hydrolytic procedure investigated, the use of ALC-PK was the most efficient procedure for the generation of hydrolysate with higher antioxidant and anti-obesity activities compared to the ALC-ACT. The fractionation of these hydrolysates on the basis of their MW by MWCO contributed to an increase in the biological activities of the hydrolysates, with the highest antioxidant and anti-obesity activities associated with the peptides of lower MW. The amino acid composition analyses performed in the hydrolysates with the highest biological activities (ALC-PK) also highlighted the influence of the relative abundance of specific amino acid residues on the biological activities of the hydrolysates. Fractions from ALC-PK with high hydrophobic amino acids, arginine, and aromatic amino acids also had high antioxidant, lipase inhibitory, and α-amylase inhibitory activities, respectively. This study contributes to the knowledge of the biological properties of novel hydrolysates from underexploited protein sources. Future studies will be necessary in order to elucidate further the structure-function relationship of these hydrolysates by further sequencing of the peptides and in silico tools that could contribute to the further understanding of these newly generated peptides.

## Figures and Tables

**Table 1:** Amino acid composition of ALC-PK hydrolysates and MWCO fractions.

Amino Acid	ALC-PK Hydrolysate	<3 kDa	3–10 kDa	10–30 kDa
Asp	9.2 ± 0.1 ^c^	12.5 ± 0.1 ^a^	11.4 ± 0.2 ^b^	11.3 ± 0.2 ^b^
Thr	3.5 ± 0.1 ^b^	4.7 ± 0.0 ^a^	4.0 ± 0.1 ^b^	3.7 ± 0.1 ^b^
Ser	4.9 ± 0.0 ^b^	5.1 ± 0.1 ^a^	4.9 ± 0.0 ^b^	4.8 ± 0.1 ^b^
Glu	17.7 ± 0.1 ^c^	20.3 ± 0.2 ^a^	19.1 ± 0.0 ^b^	17.8 ± 0.0 ^c^
Gly	4.9 ± 0.1 ^b^	6.3 ± 0.0 ^a^	5.0 ± 0.2 ^b^	5.0 ± 0.1 ^b^
Ala	4.9 ± 0.0 ^b^	5.5 ± 0.2 ^a^	4.8 ± 0.1 ^b^	4.8 ± 0.2 ^b^
Cys	6.3 ± 0.1 ^c^	6.3 ± 0.2 ^c^	7.8 ± 0.1 ^a^	6.7 ± 0.1 ^b^
Val	4.1 ± 0.0 ^c^	5.5 ± 0.1 ^a^	4.1 ± 0.0 ^c^	5.0 ± 0.1 ^b^
Met	0.9 ± 0.1 ^c^	1.6 ± 0.2 ^a^	1.0 ± 0.1 ^b^	1.0 ± 0.1 ^b^
Ile	5.2 ± 0.0 ^b^	6.7 ± 0.1 ^a^	5.4 ± 0.0 ^b^	5.2 ± 0.1 ^b^
Leu	7.4 ± 0.1 ^b^	7.2 ± 0.0 ^a^	1.1 ± 0.0 ^b^	1.1 ± 0.1 ^b^
Tyr	3.3 ± 0.0 ^b^	5.1 ± 0.2 ^a^	3.3 ± 0.0 ^b^	3.3 ± 0.1 ^b^
Phe	3.4 ± 0.1 ^c^	5.1 ± 0.0 ^a^	4.0 ± 0.2 ^b^	4.0 ± 0.0 ^b^
His	1.7 ± 0.0 ^b^	3.0 ± 0.2 ^a^	1.8 ± 0.1 ^b^	1.7 ± 0.1 ^b^
Lys	3.2 ± 0.1 ^c^	6.1 ± 0.3 ^a^	4.5 ± 0.1 ^b^	3.3 ± 0.2 ^c^
Arg	14.6 ± 0.1 ^b^	18.4 ± 0.2 ^a^	14.7 ± 0.2 ^b^	14.6 ± 0.2 ^b^
Pro	4.1 ± 0.0 ^b^	4.9 ± 0.1 ^a^	4.4 ± 0.1 ^ab^	4.1 ± 0.0 ^b^

Data on amino acid composition are expressed as g/100 g total amino acids. Data are presented as average ± standard deviation (*n* = 3). Different letters within each row represent statistical differences (*p* < 0.05) between different fractions in the content of each individual amino acid.

## Data Availability

All data generated or analyzed during this study are included in this published article.
